# MDSubSampler: *a posteriori* sampling of important protein conformations from biomolecular simulations

**DOI:** 10.1093/bioinformatics/btad427

**Published:** 2023-07-07

**Authors:** Namir Oues, Sarath Chandra Dantu, Riktaben Jigarkumar Patel, Alessandro Pandini

**Affiliations:** Department of Computer Science, Brunel University London, Uxbridge UB8 3PH, United Kingdom; Department of Computer Science, Brunel University London, Uxbridge UB8 3PH, United Kingdom; Department of Computer Science, Brunel University London, Uxbridge UB8 3PH, United Kingdom; Department of Computer Science, Brunel University London, Uxbridge UB8 3PH, United Kingdom

## Abstract

**Motivation:**

Molecular dynamics (MD) simulations have become routine tools for the study of protein dynamics and function. Thanks to faster GPU-based algorithms, atomistic and coarse-grained simulations are being used to explore biological functions over the microsecond timescale, yielding terabytes of data spanning multiple trajectories, thereby extracting relevant protein conformations without losing important information is often challenging.

**Results:**

We present MDSubSampler, a Python library and toolkit for *a posteriori* subsampling of data from multiple trajectories. This toolkit provides access to uniform, random, stratified, weighted sampling, and bootstrapping sampling methods. Sampling can be performed under the constraint of preserving the original distribution of relevant geometrical properties. Possible applications include simulations post-processing, noise reduction, and structures selection for ensemble docking.

**Availability and implementation:**

MDSubSampler is freely available at https://github.com/alepandini/MDSubSampler, along with guidance on installation and tutorials on how it can be used.

## 1 Introduction

Proteins are the workhorses of the cell as they are responsible for most of its biological functions (Branden and Tooze 2012). They undergo conformational changes to perform these functions (Henzler-Wildman and Kern 2007). Experimental studies on proteins are often complemented by molecular simulations because accessing information on dynamics across multiple time scales is generally challenging for any single technique (Orellana 2019). In particular, molecular dynamics (MD) is now being routinely used to study molecular motions at the atomistic level providing important insights into protein function (Hollingsworth and Dror 2018).

Molecular events span a vast range of timescales (picoseconds to milliseconds), requiring extensive sampling, i.e. generating multiple MD trajectories that can contain thousands to millions of conformations. Often only some of these conformations are critical to understand functional motions or are relevant for follow-up analysis. However, extracting them is challenging as they are a small fraction of the large number of recorded conformations. A most desirable solution would be to reduce simulation datasets to a manageable size avoiding information loss and retaining important subsets of conformations for subsequent analyses.

Well-established methods (Yang *et al.* 2019) are available to enhance sampling during simulations and explore the phase space more effectively. Some of these methods include techniques to reweight and extract important conformations by estimation of free energy profiles. However, there is a lack of general-purpose strategies to subsample or extract conformations *a posteriori* using statistical sampling methods which are applicable for both unbiased and biased simulations.

Built-in analysis tools included in software simulation packages have simplified but non-customizable functions to post-process protein trajectories (Abraham *et al.* 2015) and they do not offer flexible options for *a posteriori* subsampling. Implementing new methods within existing software is non-trivial as it requires advanced knowledge of the internal functionality of the specific software (Michaud-Agrawal *et al.* 2011). Alternatively, a popular approach for molecular simulation analysis is to develop *ad hoc* programmes using external libraries, such as ProDy (Bakan *et al.* 2011), MDtraj (McGibbon *et al.* 2015), or MDAnalysis (Michaud-Agrawal *et al.* 2011). Unfortunately, none of these libraries include general-purpose subsampling functions based on statistical methods. Additionally, these libraries are not designed to directly pre-process and reformat data for follow-up predictive analytics using supervised machine learning.

Here, we describe MDSubSampler, a modular Python library implementing general-purpose statistical sampling methods to extract protein conformations from biomolecular simulations data. The library facilitates the implementation of dedicated tools to extract small samples of conformations. Information loss is avoided by performing sampling under constraint that the distribution of values for relevant reference properties is consistent in the sample and original trajectory. *Ad hoc* workflows for common sampling scenarios can be easily implemented. In addition, output samples can be pre-processed into training and test sets to build predictive machine learning models, which are now more commonly used in MD studies (Kaptan and Vattulainen 2022).

MDSubSampler provides the user with three options: (i) pre-defined Python scripts and Jupyter notebooks that can be used as ‘scenario recipes’ and accommodate different user cases and applications, (ii) a command-line interface for interactive processing, and (iii) reusable library classes for software development.

## 2 Methods

**Figure 1.**
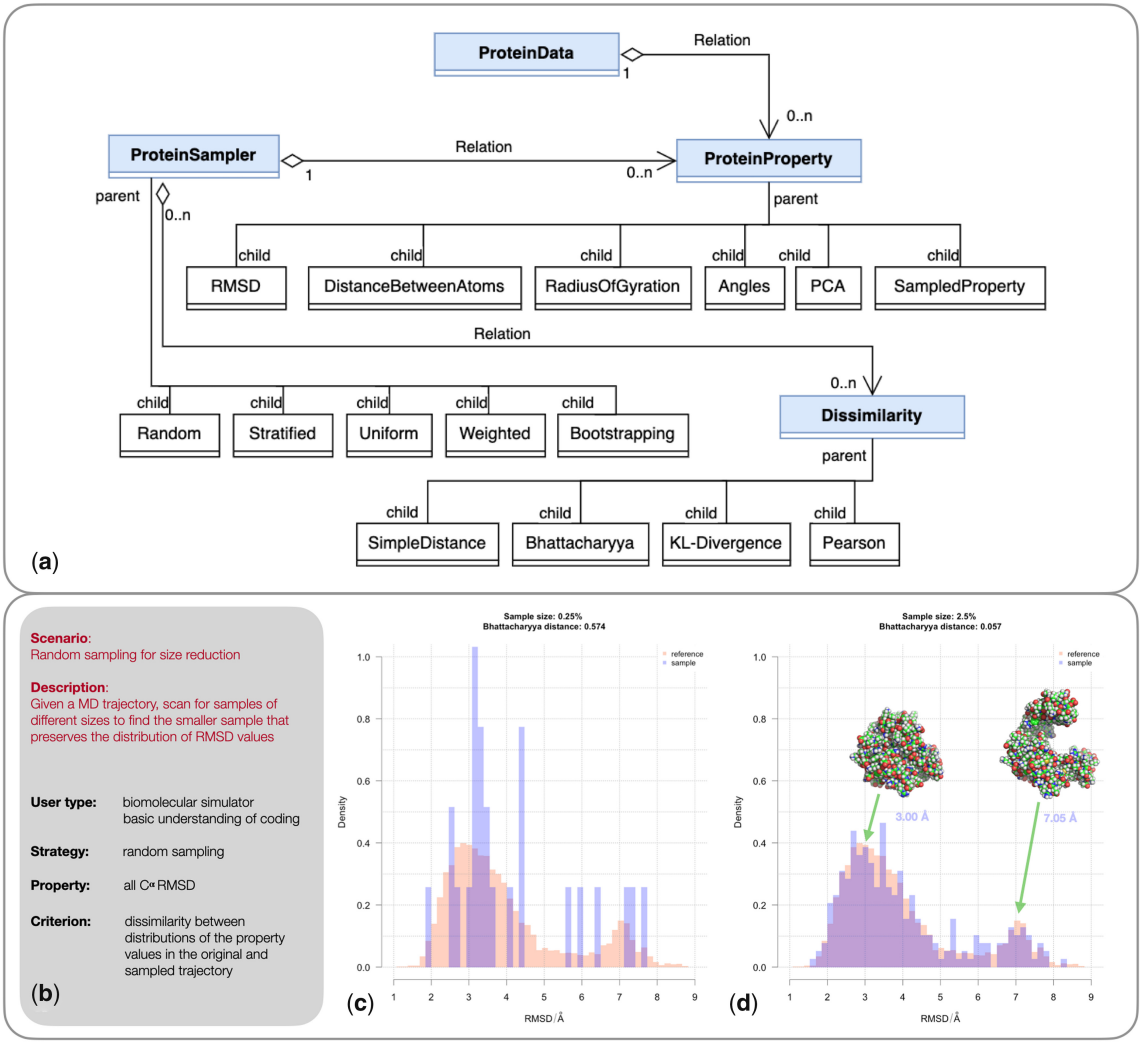
(a) Class diagram of MDSubsampler library illustrating the relationships between main classes (filled blue rectangles) and subclasses (white rectangles). The diagram depicts the multiplicity between main classes (shown as relation), where symbols indicate the number of instances of one class linked to the ones of another class, with 1 meaning exactly 1 instance, and 0.n meaning many instances. (b) Summary description of an example scenario (random sampling for size reduction) where different subsample sizes are extracted with the aim of preserving the information in the distribution of values for a reference property. (c and d) Comparison of the distributions of Root Mean Square Deviation (RMSD) over the coordinates of all C^α^ atoms in the original and subsampled trajectory for sample sizes of 0.25% and 2.5% (see Supplementary Fig. S2 for a larger range of sample sizes). The distance between the sampled and original distributions was calculated using Bhattacharyya distance: 0.574 (for 0.25%) and 0.057 (for 2.5%). A subset of 2.5% is the smallest sample for which the shape and peak’s location of the distribution of RMSD is preserved (see Supplementary Fig. S2). Example structures for an open and close conformations of adenylate kinase are reported in the top right of panel d. Distribution plots were generated with R (R Core Team 2022) and protein structure images with PyMol (Schrödinger LLC 2015).

MDSubSampler is an object-oriented Python library built on top of the popular MDAnalysis library and it is designed for subsampling large MD trajectories. MDSubSampler includes three core classes to represent: (i) protein data (‘ProteinData’); (ii) associated properties calculated over frames (‘ProteinProperty’); and (iii) automated processes to sample frames (‘ProteinSampler’). Subclasses are available to include specific geometrical properties for analysis, as well as different statistical methods for *a posteriori* sampling (see Fig. 1a).

MD trajectories can be sampled under the constraint of preserving the information for any property of interest, for example Root Mean Square Deviation (RMSD). This is done by comparing the distribution of the reference property from the sampled and original trajectory using a dissimilarity measure (‘Dissimilarity’ and relevant subclasses) and visualized using relevant plots (‘PropertyPlot’ classes) (see Fig. 1a).

MDSubSampler implements sampling strategies to extract: a specified number of random frames (‘Random’ class), an equal number of frames for ranges of a reference property (‘Uniform’), an equal number of frames for labelled subsets of the trajectory (‘Stratified’), a specified number of frames with chance proportional to user-provided frame weights (‘Weighted’) or a number of repeated random samples with replacement (‘Bootstrapping’).

The software is designed and implemented for three different user demographics: novice users with limited experience in software development can use preprepared scenario as Python scripts or Jupyter notebooks, advanced users can use a Unix-like command line interface, and scientific software developers can benefit from a set of reusable Python library classes.

MDSubSampler supports MDAnalysis input formats and returns subsampled outputs for properties and trajectories for further analysis, including training of predictive machine learning models.

Methods details on data preparation, sampling, and output generation are reported in the Supplementary Data.

## 3 Results

Adenylate kinase has been selected as an example protein for development and testing as it has a clearly defined functional conformational change, as well as availability of reference experimental structure for validation (Ping *et al.* 2013). To demonstrate potential uses of MDSubSampler, we present three use-case scenarios: random sampling for size reduction, pocket sampling for ensemble docking, and sampling by most frequently observed conformations.

*Random sampling for size reduction*: This scenario demonstrates how to extract the smallest random subsample of trajectory frames preserving the distribution of a reference geometrical property, i.e. RMSD using C^α^ atoms, with random sampling over a range of sample sizes provided by the user (Fig. 1b). The Bhattacharyya distance (Bhattacharyya 1933) between RMSD distributions from reference and sampled trajectories is calculated (Fig. 1c and d) to identify the smallest sample size.

*Pocket sampling for ensemble docking*: This scenario demonstrates how to extract a subsample of desired size that equally represents the range of values for the opening of a pocket, using uniform random sampling as it is ideal for this purpose. The subsample can be used for pocket analysis or ensemble docking. The opening of the pocket was monitored using the C^α^ atoms RMSD of the lid (residue 120–160) (Supplementary Fig. S3).

*Sampling by most frequently observed conformations*: This scenario demonstrates how to extract a subsample of frames by randomly selecting frames according to the frequency of a reference property, i.e. RMSD to a target conformation (Supplementary Fig. S4). Independently calculated measures, i.e. cavity volumes or energy rescoring, can also be used as reference property as along as a value is available for each trajectory frame.

Output trajectories from MDSubSampler can be reshaped as tensors and saved in binary format for input to Python scripts for machine learning.

Summary description of the workflows, detailed results for each scenario and example of output preparation for supervised machine learning are reported in the Supplementary Data.

## 4 Conclusions

MDSubSampler provides a set of Python tools for users with different levels of programming experience. The tool addresses the need for automated strategies to extract subsets of frames from MD trajectories using statistical methods. Extracted subsets preserve or better describe the range and distribution of values in a time-dependent property of the system. The subset of frames can be saved in suitable binary formats for further analyses and can be reformatted to be directly read as input for machine learning methods.

MDSubSampler is designed to offer an *a posteriori* approach to sampling that is independent from how the original trajectory was generated. This provides a general-purpose tool, but the library can also be extended to implement *ad hoc* workflows guided by specific sampling strategies, i.e. by using energetic properties or kinetic information.

MDSubSampler complements traditional clustering methods through the flexibility of statistical sampling guided by reference properties. Indeed, workflows can easily be designed with MDSubSampler to apply clustering in combination with statistical sampling.

MDSubSampler facilitates tasks related to simulations post-processing, subsampling for noise reduction, and structure selection for ensemble docking. Using MDSubSampler classes, Python scripts can be easily developed to implement automated workflow for analysis, dataset preparation, and training of machine learning models.

## Supplementary Material

btad427_Supplementary_Data

## Data Availability

A sample of the trajectory data is included in the GitHub repository. The data underpinning this publication can be accessed from Brunel University London’s data repository under CC BY licence: https://doi.org/10.17633/rd.brunel.c.6620539.
