# Supplementary material for: MDSubSampler: a posteriori sampling of important protein conformations from biomolecular simulations
Source: Bioinformatics. 2023 Jul 7;39(7):btad427. doi: 10.1093/bioinformatics/btad427 (PMC10348832; doi:10.1093/bioinformatics/btad427)
Supplement: btad427_Supplementary_Data [file btad427_supplementary_data.pdf]

Supplementary Information

1 Methods

Software design. MDSubSampler was developed as a pip installable object-oriented Python library (see Figure S1). The library is built on top of the trajectory and analysis classes from MDAnalysis (Michaud-Agrawal *et al.*, 2011) and it is designed to provide a set of classes to represent and process samples of trajectory frames. The core class is **ProteinData** that encapsulates data on protein structure, protein topology and an associated molecular dynamics simulation (MD) trajectory. Properties over frames can be read from a user supplied file or calculated on-the-fly and MDSubSampler provides a set of classes inheriting from the superclass **ProteinProperty**. ProteinData objects have a dictionary attribute to hold references to the associated ProteinProperty objects. These objects record the frame indices for each value, providing an easy way to map property values to trajectory frames. Samples can be extracted from ProteinData trajectories using a choice of subsampling strategies implemented by subclasses of **ProteinSampler**: random sampling, uniform random sampling, stratified sampling, weighted sampling and bootstrapping. ProteinSampler objects are created with reference to a ProteinProperty object and return the subsampled property objects. Both property values and associated trajectory frames can be saved to file. Subsampled and original sets of property values can be compared using **Dissimilarity** objects that implement a set of widely used distance measures between statistical distributions: Bhattacharyya distance (Bhattacharyya, 1933), Kullback-Leibler divergence (Kullback and Leibler, 1951) and Pearson correlation (Pearson, 1895) distance.

Software implementation. MDSubSampler was developed using git version control system. The project and documentation are hosted on GitHub (https://github.com/alepandini/MDSubSampler). MDSubSampler is an installable library and contains a set of classes (see Software Design above) to develop software solutions to sample frames from MD trajectories. A command line Python script is provided to access all the main options and three example scenarios are included in Python script and Jupyter notebook format. A cookbook directory is available in the repository where recipes for advanced workflows will be deposited.

Example case: protein system preparation. The Adenylate kinase (ADK) structure (PDBID: 4AKE) was download from the Protein Databank (PDB) (Berman et al. , 2003). System preparation and simulations were done using Amber 20 with ff14SB force field (Case et al., 2020). The protein was immersed in a truncated octahedron water box with minimum distance between solute and box boundaries of 10 Å and was solvated with TIP3P (Jorgensen *et al.*, 1983) water molecules. Four sodium ions were added to neutralize the protein charge. Energy minimization was performed in two steps: first 2500 cycles of steepest descent followed by 2500 cycles conjugate gradient descent were carried out with backbone restraints; then the system was relaxed for 2500 cycles steepest descent and 2500 cycles conjugate gradient descent without any constraints. The non-bonded cut-off for both steps was 8 Å.

Example case: MD simulations. The minimized system was then equilibrated in two steps: a) the system was heated for 100 ps at constant volume and temperature (NVT) using a Langevin thermostat (Loncharich *et al.*, 1992) b) then the system was simulated at constant pressure and temperature (NPT) for 250 ps. The Berendsen barostat (Postma *et al.*, 1984) was used with pressure coupling time of 0.5 ps. The temperature coupling time was set to 1.0 ps. Long-range electrostatic interactions were treated by the particle mesh Ewald (PME) (Darden *et al.*, 1999) method under periodic boundary conditions with the non-bonded cut-off distance of 8 Å. After equilibration a production simulation of 1 μs time with time step of 2 fs was completed.

2 Results

Random sampling for size reduction. The RMSD over C^{α} atoms of the protein was calculated for each frame with respect to the reference structure provided as input. All structures were superimposed to the reference structure before RMSD calculation. The distribution of the RMSD values clearly indicates the presence of two conformations consistent with a close and open arrangement of the ADK lid domain (see Figure 1c-d). Random samples of frames were extracted for given subsets (0.25%, 0.5%, 1%, 2.5%, 5%, 10%, 20%, 25%, 50%) of the original trajectory. The associated distributions of the RMSD values (see Figure 1c-d and S2) were compared to the original trajectory using Bhattacharya distance. The smallest sample that preserves the bimodal distribution in the original trajectory is of size 2.5% of the original trajectory. This sample was automatically returned by the scenario, the associated RMSD values were saved as text file and the trajectory frames were saved in a binary xtc file.

Pocket sampling for ensemble docking. The RMSD over C^{α} atoms of the lid (residue 120-160) was calculated for each frame with respect to the reference structure supplied as an input. All structures were superimposed to the reference structure before RMSD calculation. The range of RMSD values from the close to the open conformation (as represented by the extreme values of lid RMSD) was divided in 200 intervals. For each interval 10% of total number of structures (i.e., full trajectory) were randomly selected. The resulting collection of frames equally samples the range of possible opening for the protein binding site (see Figure S3). This sample was automatically returned by the scenario, the associated RMSD values were saved as text file and the trajectory frames were saved in a binary xtc file.

Sampling by most frequently observed conformations. The RMSD over C^{α} atoms of the lid was calculated for each frame with respect to the reference structure supplied as an input. All structures were fit to the reference structure before RMSD calculation. The range of values of RMSD were discretized in 100 bins. Frequency counts were then recorded for each bin and used as weight for each frame. 10% of the total number of frames from the original trajectory were selected by weighted random sampling. The resulting collection of frames contained random structures selected from the most frequently observed conformations. This generated an enrichment of the close conformations compared to unweighted random sampling (see Figure S4).

Input preparation for machine learning. The trajectory frames from the output subsamples were also automatically saved as NumPy arrays in binary files. The coordinate matrix was reshaped from a 3D array (dimensions: number of atoms, cartesian coordinates, number of frames) to a 2D array (dimensions: number of frames, number of atoms x cartesian coordinates). This output format provides two advantages: a) a compressed file format easily read in input by machine learning libraries; b) a tabular format where frames can be considered as input instances and coordinates can be considered as features. The trajectory frames from the output subsample can be automatically split into training and test set files for machine learning input.

References

Berman,H. *et al.* (2003) Announcing the worldwide Protein Data Bank. *Nat Struct Biol*, **10**, 980.

Bhattacharyya,A. (1933) On a Measure of Divergence between Two Multinomial Populations. *The Indian Journal of Statistics*, **7**, 401–406.

Case Ross C Walker,D.A. and Roitberg Kenneth Merz Pengfei Li,A.M. Amber 2020 Reference Manual Principal contributors to the current codes.

Darden,T. *et al.* (1999) New tricks for modelers from the crystallography toolkit: the particle mesh Ewald algorithm and its use in nucleic acid simulations. *Structure*, **7**, R55–R60.

Jorgensen,W.L. *et al.* (1983) Comparison of simple potential functions for simulating liquid water. *J Chem Phys*, **79**, 926–935.

Kullback,S. and Leibler,R.A. (1951) On Information and Sufficiency. *The Annals of Mathematical Statistics*, **22**, 79–86.

Loncharich,R.J. *et al.* (1992) Langevin dynamics of peptides: the frictional dependence of isomerization rates of N-acetylalanyl-N’-methylamide. *Biopolymers*, **32**, 523–535.

Michaud-Agrawal,N. *et al.* (2011) MDAnalysis: A toolkit for the analysis of molecular dynamics simulations. *J Comput Chem*, **32**, 2319–2327.

Pearson,K. (1895) VII. Note on regression and inheritance in the case of two parents. *Proceedings of the Royal Society of London*, **58**, 240–242.

Postma,J.C. *et al.* (1984) Molecular dynamics with coupling to an external bath. *Studies in Molecular Dynamics. I. General Method The Journal of Chemical Physics*, **81**, 234505.

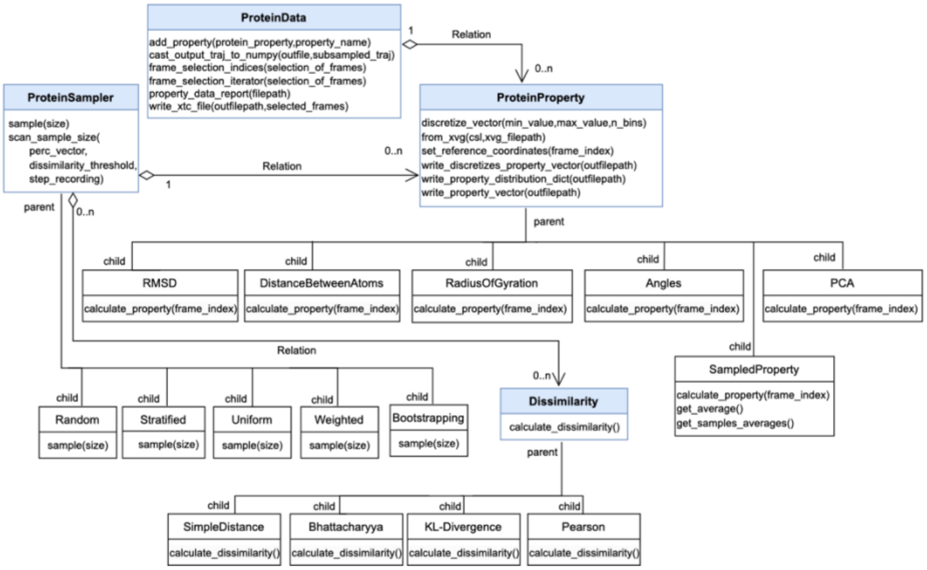

Figure S1. Class diagram of MDSubSampler library illustrating the relationships between main classes (blue) and subclasses (white). The diagram depicts the multiplicity between main classes (shown as relation), where symbols indicate number of instances of one class linked to number of instances of another class, with 1 meaning exactly 1 instance, and 0...n meaning many instances.

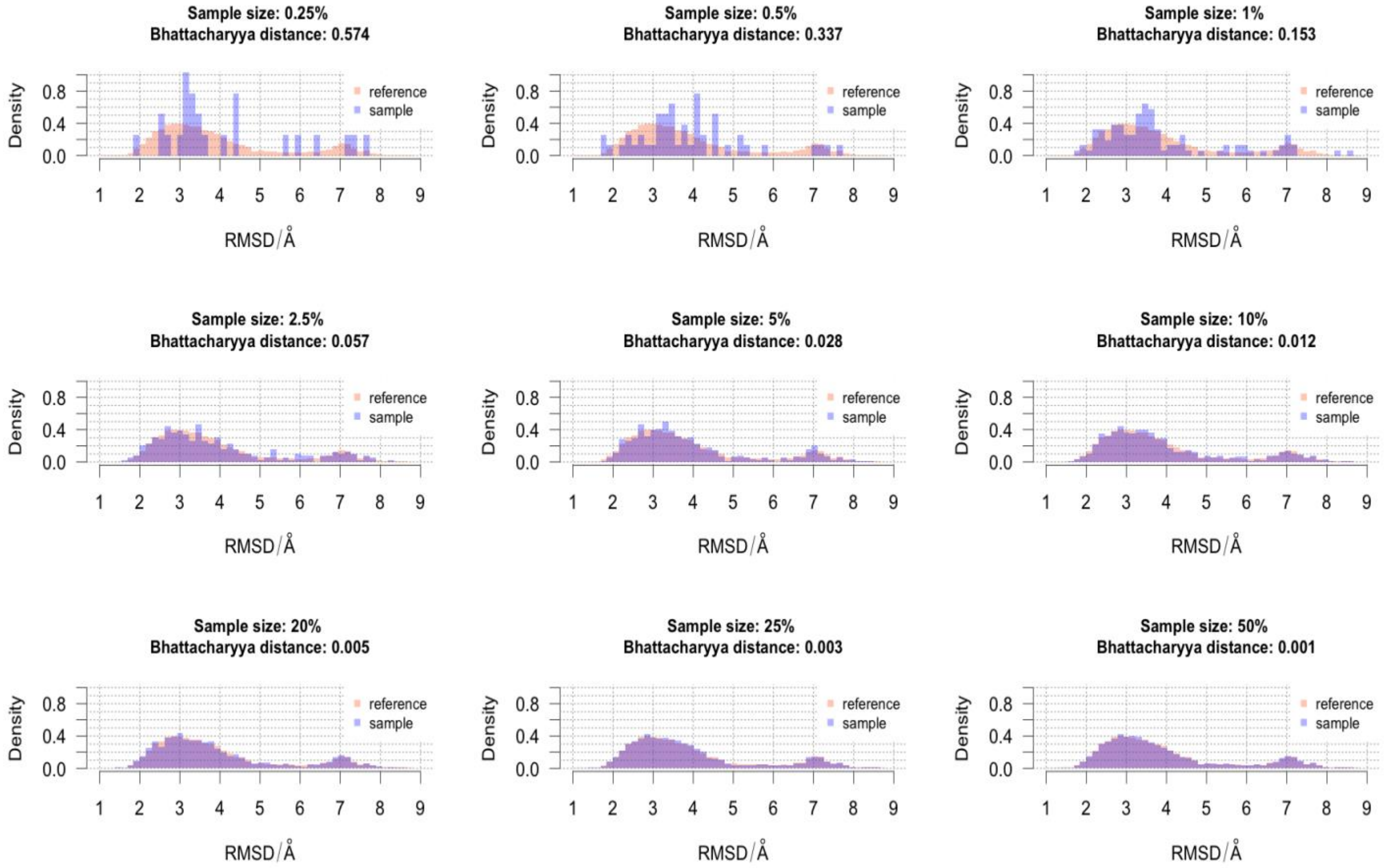

Figure S2. Summary results for a scenario of “Random sampling for size reduction”: comparison of the distributions of Root Mean Square Deviation (RMSD) over the coordinates of all C$^{\alpha}$ atoms in the original and subsampled trajectory for different sample sizes ((0.25%, 0.5%, 1%, 2.5%, 5%, 10%, 20%, 25%, 50%). The distance between the sampled and original distributions was calculated using Bhattacharyya distance. A subset of 2.5% is the smallest sample for which the shape and peaks location of the distribution of RMSD is preserved.

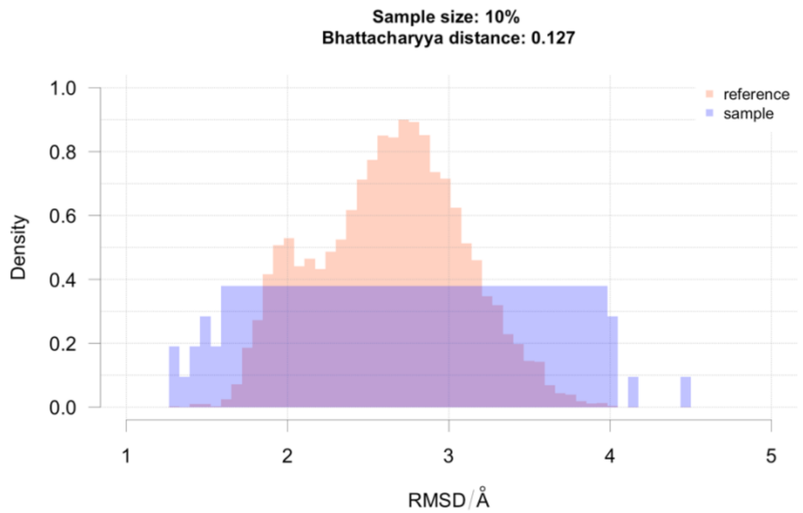

Figure S3. Summary result for a scenario of “Uniform sampling of pocket opening for ensemble docking”. RMSD over the C$^{\alpha}$ atoms of ADK lid was calculated for all frames. The range of RMSD values from closed to open state was divided in 200 intervals and for each interval a random sample of 10% of frames was selected. This set of frames equally samples the range of possible opening for binding site of protein. The distance between the sampled and original distributions was calculated using Bhattacharyya distance.

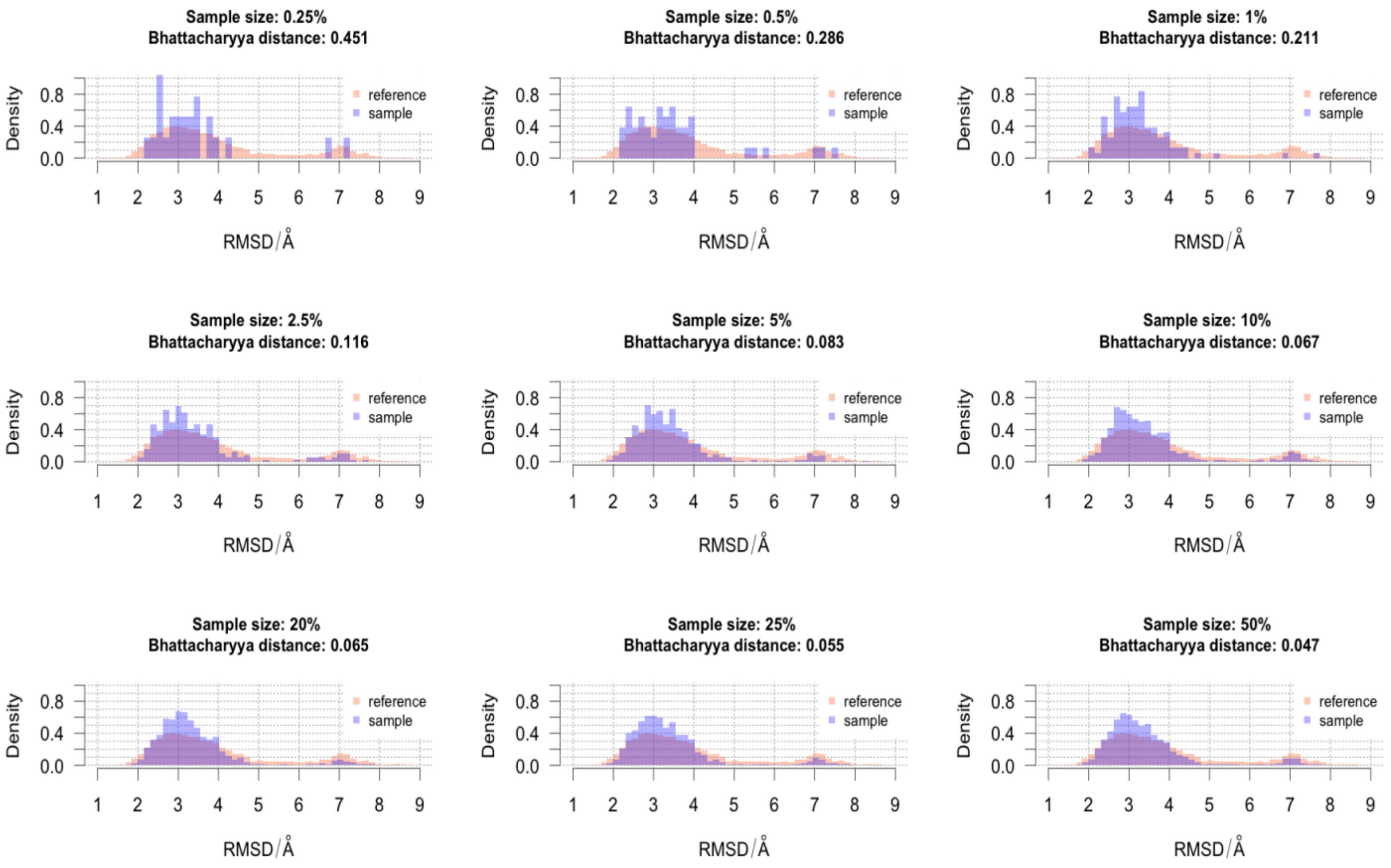

Figure S4. Summary results for a scenario of "Weighted sampling of pocket openings for ensemble docking". RMSD values for ADK lid opening were calculated for each frame. The range of values were then discretized in 100 bins and frequency counts were recorded for each bin and used as weight for each frame. The resulted set of frames was 10% of the original trajectory and was extracted by weighted random sampling. This set contains random structures selected from the most frequently observed conformations in the original trajectory. This generates an enrichment of the close conformations compared to unweighted random sampling.
